# The mitochondrial genomes of Atlas Geckos (*Quedenfeldtia*): mitogenome assembly from transcriptomes and anchored hybrid enrichment datasets

**DOI:** 10.1080/23802359.2017.1339212

**Published:** 2017-06-14

**Authors:** Mariana L. Lyra, Ulrich Joger, Ulrich Schulte, Tahar Slimani, El Hassan El Mouden, Abdellah Bouazza, Sven Künzel, Alan R. Lemmon, Emily Moriarty Lemmon, Miguel Vences

**Affiliations:** a Departamento de Zoologia, Instituto de Biociências, UNESP – Universidade Estadual Paulista, Rio Claro, Brazil;; b Staatliches Naturhistorisches Museum, Braunschweig, Germany;; c Büro für Faunistische Gutachten, Borgholzhausen, Germany;; d Faculty of Sciences, Biodiversity and Ecosystem Dynamics Laboratory, Cadi Ayyad University, Marrakech, Morocco;; e Max Planck Institute for Evolutionary Biology, Plön, Germany;; f Department of Scientific Computing, Florida State University, Dirac Science Library, Tallahassee, FL, USA;; g Department of Biological Science, Florida State University, Tallahassee, FL, USA;; h Zoological Institute, Technical University of Braunschweig, Braunschweig, Germany

**Keywords:** Squamata, Gekkota, Sphaerodactylidae, *Quedenfeldtia moerens*, *Quedenfeldtia trachyblepharus*, Morocco

## Abstract

The nearly complete mitogenomes of the two species of North African Atlas geckos, *Quedenfeldtia moerens* and *Q. trachyblepharus* were assembled from anchored hybrid enrichment data and RNAseq data. Congruent assemblies were obtained for four samples included in both datasets. We recovered the 13 protein-coding genes, 22 tRNA genes, and two rRNA genes for both species, including partial control region. The order of genes agrees with that of other geckos.

The genus *Quedenfeldtia* (Reptilia, Squamata, Sphaerodactylidae) comprises two species of diurnal geckos, *Quedenfeldtia moerens* (Chabanaud 1916) and *Quedenfeldtia trachyblepharus* (Boettger 1874). Both are endemic to Morocco (Arnold 1990), and sometimes named Atlas day geckos due to their occurrence only in the Atlas Massif.

The two species of *Quedenfeldtia* are altitudinally segregated along the slopes of the Atlas Massif: *Q. moerens* occurs from sea level to 3000 m above sea level, whereas *Q. trachyblepharus* is restricted to highland sites, occurring from 1200 to 4000 m a.s.l. (Barata et al. 2012), and anatomical differences support substantially separated evolutionary histories of the two species (Arnold 1990). Genetically, the two species were found reciprocally monophyletic, but each with substantial genetic variation and composed of distinct subclades (Barata et al. 2012). Here, we report near-complete mitochondrial genomes of four individuals of *Q. trachyblepharus* and two individuals of *Q. moerens* from different localities in Morocco.

Six samples were processed for anchored hybrid-enrichment by the Center for Anchored Phylogenomics at Florida State University (www.anchoredphylogeny.com) with a method for anchored hybrid enrichment analysis (Lemmon et al. 2012). Genomic DNA was sonicated to a fragment size of ∼300–700 bp and libraries prepared and indexed following the protocol of Meyer and Kircher (2011) with minor modifications. Equal quantities of indexed samples were pooled and enrichments performed with probes designed for anchored loci from Amniotes (Prum et al. 2015; Ruane et al. 2015; Tucker et al. 2016). Sequencing was carried out on an Illumina HiSeq2500 sequencer. Voucher specimens were not collected because specimens were destructively sampled for transcriptomic analysis (see below). Tissues and extracted DNA of all samples are kept at the Staatliches Naturhistorisches Museum in Braunschweig, under the field numbers given in the following: *Quedenfeldtia moerens*: UJLAC 39 from Amassine (Jebel Siroua; lat 30.831, long −7.608, 2187 m); UJLAC 40 from 10 km W Tafraoute (29.658, −9.053, 1003 m); *Quedenfeldtia trachyblepharus*: UJLAC 12 and 41 from Oukaimeden (31.203, −7.869, 2740 m); UJLAC 28 from Jebel Toubkal (31.074, −7.933, 2971 m); UJLAC 42 from a site in the High Atlas (30.979, −7.711, 2391 m).

Mitochondrial genomes of four of the individuals (UJLAC 39–42) were also assembled from transcriptomic (RNAseq) data. Samples of a mix of different organs (muscle, liver, skin, heart, lungs) were taken from freshly sacrificed specimens, immediately stored in RNAlater and preserved at −80 °C. Extraction from 100 mg of tissue of each sample was carried out using a trizol protocol. RNA was prepared for sequencing following the Illumina TruSeq mRNA protocol and sequenced on the Illumina NextSeq (2 × 75 bp paired-end) platform.

We assembled mitogenomes from the quality-trimmed Illumina reads of either DNA (anchored hybrid enrichment) or RNA (RNAseq). For RNAseq, we first normalized raw data to an average depth of 100× using BBNorm (BBMap/BBTools; http://sourceforge.net/projects/bbmap/) to reduce computational effort. For assembly we used MIRA v4.0 (Chevreux et al. 1999) and MITObim v1.8 (Hahn et al. 2013), with default parameters, and using the reference mitogenome of *Teratoscincus roborowskii* (KP115216) as initial seed. We manually verified assemblies in Geneious software v.6 (Biomatters Auckland, New Zealand) to evaluate the coverage and quality of each mitochondrial element, and coded positions with coverage lower than 4 as ambiguous (‘N’). Preliminary annotation of each sequence was done using the mitochondrial genome annotation server MITOS (Bernt et al. 2013), tRNA sequences validated with tRNAscan-SE (Lowe and Chan 2016), and the annotation manually validated by comparison to the *Teratoscincus* mitochondrial genome. New sequences were submitted to GenBank (accession numbers KY996812–KY996817). Sequences were aligned to published complete or near-complete mitochondrial genomes of gekkotan lizards with MAFFT v.7 (Katoh and Standley 2013). Maximum Likelihood phylogenetic inference was performed in RAxML V 7.2.7 (Stamatakis 2006) using the GTRGAMMA substitution model and rapid bootstrap heuristics with 100 pseudo-replicates, as implemented in the CIPRES Science Gateway (Miller et al. 2010).

We assembled nearly complete mitogenomes both from the DNA and RNA datasets as by-products besides the nuclear DNA or mRNA targets. Although we recovered more mitochondrial reads with RNAseq, the raw datasets from the anchored hybrid enrichment allowed us to recover more complete mitogenomes, particularly in intergenic regions as expected. This result is probably also due to read length differences (150 bp for anchored hybrid enrichment and 75 bp for RNAseq). The only exception was sample UJLAC41, for which we could not retrieve the complete sequences of ND5 and ND6 from the anchored hybrid-enrichment data.

The mitogenomes of *Q. moerens* and *Q. trachyblepharus* have the typical composition found for other lizards, consisting of 13 protein-coding, two ribosomal RNA (rRNA), 22 transfer RNA (tRNA) genes, and the control region (D-loop; not completely sequenced). The arrangement of genes is consistent with other gekkotan lizards. All coding genes are encoded on the heavy strand, except ND6 and eight tRNA genes. The protein-coding genes are normally initiated with the codon ATG, but also GTG, ATA, ATT, and ATG, and with five types of stop codons (TAA, TAG, AGA, TA−, T–). The control region (D-loop) is located between tRNA-Pro and tRNA-Phe and just a few non-coding regions exist between genes. Overlaps in adjacent coding genes were found between ATP8/ATP6 (10 bases), ATP6/COIII (one base), ND4L/ND4 (seven bases), and ND5/ND6 (three bases).

As already assessed in previous studies (Barata et al. 2012), both species of *Quedenfeldtia* are characterized by substantial genetic variation among populations. In our sampling, the uncorrected pairwise distance (p-distance) between the two species for (a) the whole mitochondrial genome, (b) the barcoding region of the COI gene, and (c) the 3′-terminal 648 bp of the 16S rRNA gene was of 13.5–14.%, 13.1–15.7%, and 6.3–7.1%, respectively. The maximum intraspecific distances were 10.1%, 9.3%, and 3.4%, respectively, for *Q. trachyblepharus*, and 7.5%, 8.8%, and 3.6% for *Q. moerens*.

In the most comprehensive analyses of gecko relationships to date (Gamble et al. 2012; Pyron et al. 2013), *Quedenfeldtia* was recovered as sister group of *Aristelliger* within the Sphaerodactylidae, and the clade of these two genera was sister to a clade comprising *Euleptes*, *Saurodactylus*, and *Teratoscincus*. The sister-group relationship between *Quedenfeldtia* and *Aristelliger* has also been recovered in other phylogenetic analyses (e.g. Gamble et al. 2008, 2011) and is remarkable because *Aristelliger* is a New World genus, occurring in the Caribbean region, and probably has dispersed to this region in ancient times, in the Late Mesozoic or Early Tertiary, similar to several other gecko clades (Gamble et al. 2011). The phylogenetic tree inferred using complete and partial mitogenomes (Figure 1) is largely consistent with these previous analyses in that it recovers *Quedenfeldtia* as sister group of *Teratoscincus*, the only other species of the family Sphaerodactylidae with available mitogenomic data.

**Figure 1.**
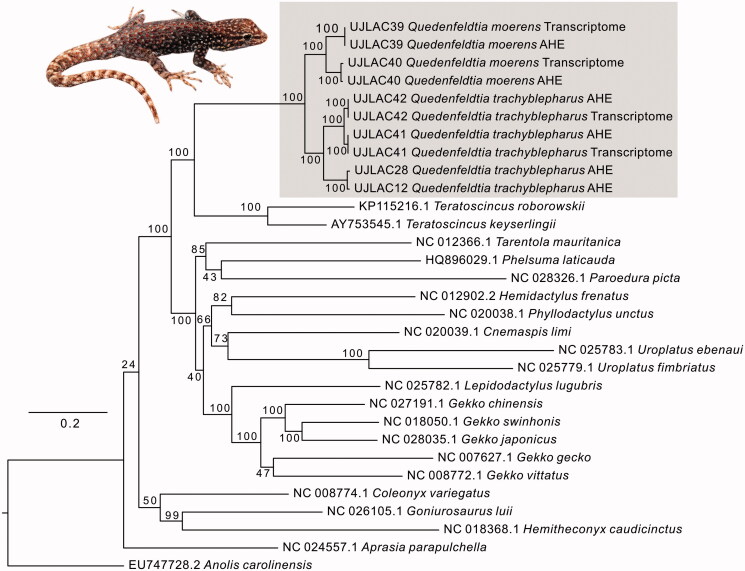
Maximum-likelihood tree constructed under the GTR model. Bootstrap support is shown at nodes. Grey box shows *Quedenfeldtia* sequences newly obtained for this study; inset picture shows representative *Q. trachyblepharus* from Oukaimeden.
